# Intracellular Development of Resident Cardiac Stem Cells: An Overlooked Phenomenon in Myocardial Self-Renewal and Regeneration

**DOI:** 10.3390/life11080723

**Published:** 2021-07-21

**Authors:** Galina Belostotskaya, Dmitry Sonin, Michael Galagudza

**Affiliations:** 1 Sechenov Institute of Evolutionary Physiology and Biochemistry, Russian Academy of Sciences, 194223 St. Petersburg, Russia; 2 Almazov National Medical Research Centre, Institute of Experimental Medicine, 197341 St. Petersburg, Russia; sonin_dl@almazovcentre.ru (D.S.); galagudza_mm@almazovcentre.ru (M.G.)

**Keywords:** cardiac stem cells (CSCs), cardiomyocytes (CMs), “cell-in-cell structures” (CICSs), transitory amplifying cells (TACs), myocardial ischemia, regeneration, cardiomyogenesis

## Abstract

At present, the approaches aimed at increasing myocardial regeneration after infarction are not available. The key question is the identity of cells capable of producing functional cardiac myocytes (CMs), replenishing those lost during ischemia. With identification of resident cardiac stem cells (CSCs), it has been supposed that this cell population may be crucial for myocardial self-renewal and regeneration. In the last few years, the focus has been shifted towards another concept, implying that new CMs are produced by dedifferentiation and proliferation of mature CMs. The observation that CSCs can undergo development inside immature cardiac cells by formation of “cell-in-cell structures” (CICSs) has enabled us to conclude that encapsulated CICSs are implicated in mammalian cardiomyogenesis over the entire lifespan. Earlier we demonstrated that new CMs are produced through formation of CSC-derived transitory amplifying cells (TACs) either in the CM colonies or inside encapsulated CICSs. In this study, we described the phenomenon of CSC penetration into mature CMs, resulting in the formation of vacuole-like CICSs (or non-encapsulated CICSs) containing proliferating CSCs with subsequent differentiation of CSC progeny into TACs and their release. In addition, we compared the phenotypes of TACs derived from encapsulated and non-encapsulated CICSs developing in immature and mature CMs, respectively.

## 1. Introduction

The high rate of chronic heart failure development after massive cardiomyocyte (CM) death in myocardial infarction (MI) has been traditionally attributed to very low regenerative capacity of mammalian myocardium. However, identification of resident cardiac stem cells (CSCs) of c-kit^+^, Sca-1^+^ and Islet-1^+^ types [1,2,3] and the subsequent verification of their cardiomyogenic potential have contributed to the hypothesis that CSCs are essential to the process of myocardial self-renewal and regeneration [4,5]. This idea has inspired numerous experimental and also clinical studies testing the effects of CSC administration into the myocardium after infarction [6,7]. This approach has been associated with minimal improvement in left ventricular function, which is thought to be related to paracrine cardioprotective and anti-remodeling signaling. Therefore, the ability of transplanted CSCs to build up new contracting CMs has been questioned. The lack of convincing data on the involvement of CSCs in in vivo cardiomyogenesis has led to a conclusion that the adult mammalian myocardium contains a minimal, if any, amount of c-kit^+^ CSCs [8]. Together with the results of Porrello et al. [9] on the cessation of CM division in mammalian heart at the end of the first week of life, these data have shifted the emphasis away from the concept that CSCs are crucial for cardiac regeneration toward the hypothesis that cardiomyogenesis could be achieved by the stimulation of mature CM proliferation. According to this prevailing view, partially dedifferentiated mature CMs are able to enter the cell cycle and produce new CMs [10,11]. It should be noted, however, that other groups continue to explore the role of c-kit^+^ CSCs in cardiomyogenesis in order to prompt myocardial repair after injury by virtue of stimulating CSC differentiation into CMs [12,13]. In particular, our group earlier demonstrated the existence of two pathways for cardiomyogenesis involving CSCs in adult mammalian species: (1) through the formation of CSC-derived cell colonies, giving rise to transitory amplifying cells (TACs), which further differentiate into CMs [14]; and (2) through intracellular development of CSCs with the formation of “cell-in-cell structures” (CICSs) [15,16]. As far as the latter pathway is considered, it has been demonstrated that CSCs are able to penetrate into immature rodent CMs through invagination of sarcolemma, thereby forming CICSs characterized by the D ≤ 30 µm and prominent capsule containing 3–5 pores that opened into the host-cell sarcoplasm. Based upon the detailed description of CICS morphology and demonstration of their rupture followed by the release of numerous TACs, we have suggested that this process might be implicated in myocardial self-renewal and regeneration [17]. The key question is whether these phenomena are observed in the settings of myocardial ischemia–reperfusion injury and permanent coronary artery ligation. Furthermore, one cannot exclude the possibility that the density of CICSs, as well as the qualitative characteristics of their development, might be different in infarcted vs. intact myocardial tissue. Therefore, we thought to investigate the distribution of CICSs in different areas of the heart in adult rats subjected either to myocardial ischemia–reperfusion or permanent ischemia. Our data confirmed more intensive release of TACs from pre-existing encapsulated CICSs during 1–5 days after permanent coronary artery occlusion. In addition, we have demonstrated that mature CMs with fully developed sarcomeric apparatus contain vacuole-like, membrane-bound CICSs releasing TACs which appear to have undergone more advanced cardiomyogenic differentiation compared to those released from encapsulated CICSs. These data may have important applications for stimulation of myocardial regeneration in the early phase after myocardial infarction.

## 2. Methods

**Ethical Statement.** All procedures were performed in accordance with the Guide for the Care and Use of Laboratory Animals (NIH publication No. 85-23, revised 2011) and the European Convention for the Protection of Vertebrate Animals used for Experimental and other Scientific Purposes. The Institutional Animal Care and Use Committee at Almazov Centre approved the study protocol.

**Animals.** Male, 260–320 g, SPF Wistar rats (Pushchino, Moscow region, Russian Federation) were used throughout the experiments. The animals were maintained under a 12/12-h light/dark cycle with free access to food and water.

**Experimental Design.** The animals were randomly allocated to 3 groups: myocardial ischemia–reperfusion (MIRI, *n* = 5), permanent coronary artery occlusion (PCAO, *n* = 5), and sham-operated animals (SHAM, *n* = 5).

**Myocardial Ischemia–Reperfusion and Permanent Ischemia Model.** Anesthesia was induced by placing the animal in a chamber filled with 5% isoflurane, using low-flow gas apparatus (SomnoSuite, Kent Scientific, Torrington, CT, USA). After induction, the animals were tracheotomized and ventilated (SAR-830P; CWE, Inc., Ardmore, PA, USA), using gas containing 2% isoflurane, 35% oxygen, and 63% room air, with a tidal volume of 2 mL/100 g and an approximately 60 breaths per minute rate. Body temperature was maintained at 37.0 ± 0.5 °C by a feedback-controlled heating pad (TCAT-2LV; Physitemp Instruments Inc., Clifton, NJ, USA). The left carotid artery and right femoral vein were cannulated for measuring mean arterial pressure (MAP) and drug injection, respectively. Lead II of the electrocardiogram was monitored for registering heart rate (HR) and arrhythmias. After 10 min of stabilization, left thoracotomy was performed. A 6.0 polypropylene thread was placed around the left coronary artery, and the ends passed through a polyethylene tube as an occluder. After surgical preparation, the animals were subjected either to 30 min coronary artery occlusion followed by 180 min reperfusion (MIRI group) or permanent coronary occlusion for 1–5, 7, or 14 days (PCAO group). Exclusion criteria were MAP < 50 mmHg and/or HR < 300 at any time point during the experiment. The animals were then euthanized by the overdose of isoflurane, followed by heart excision and preparation for cell experiments.

**Isolation of cardiac cells for ex vivo experiments.** Dissected myocardial fragments of hearts were enzymatically dissociated into a single cell and small fragment suspension as previously described [15]. Briefly, the hearts were excised and rinsed in the Ringer’s solution (pH 7.4) consisting of 146 mM NaCl, 5 mM KCl, 2 mM CaCl2, 1 mM MgCl2, 11 mM glucose, and 10 mM HEPES. After mincing and incubation in the same solution with the addition of 1 mg/mL collagenase IA (Sigma-Aldrich, Burlington, MA, USA) and 0.12% trypsin (FLUKA, Sigma-Aldrich) at 37 °C for 30 min, the suspension of gentle pipetted cells was centrifuged at 400× *g* for 10 min. Gentle pipetting and centrifugation were used to preserve the integrity of myocardial CSC-derived colonies and CICSs with TACs. The enzyme-free cell suspension was stained, using antibodies, followed by suspending the cells between the slide and cover slip.

The number of encapsulated TAC-containing CICSs was estimated in the suspension of myocardial cells. Encapsulated CICSs were quantified in each of three groups in triplicate. It seems important to note that the methodology used is our know-how based on quantification of encapsulated CICSs in the suspension of immunocytochemically labeled cells. Since the weight of initial myocardial samples varied from 50 to 120 mg, the number of CICSs is expressed per 100 mg of tissue.

This particular technique of cell isolation was used because we aimed to maintain the integrity of specific cellular structures, including CSC-derived colonies and CICSs containing TACs, which are not identifiable with other approaches. Given rare occurrence of CSC-derived colonies and CICSs (1 colony and 1 CICS per 100,000 CMs), we were as yet unable to identify and describe these structures by using FACS analysis, or confocal or electron microscopy of myocardial tissue specimens. Taking this into account, the used technique of ex vivo characterization of freshly isolated mammalian myocardial cells seems to be optimal for identification of previously unknown CICSs with TACs and CSC-derived colonies of different size and maturity.

**Immunocytochemistry.** After rinsing with PBS and fixation for 20 min in 2.5% paraformaldehyde at room temperature, the cells were permeabilized with 0.25% Triton-X100 for 10 min. For immunostaining, we used primary mouse anti-Islet-1 antibodies (Abcam, Cambridge, UK) and mouse antibodies to sarcomeric alpha-actinin (Abcam), alpha-sarcomeric actin (Sigma-Aldrich), and cardiac troponin T (Abcam) pre-conjugated with Alexa 532, 546, 568, 594, or 647 according to Zenon technology (Invitrogen). Commercially available FITC-conjugated anti-c-kit (Abcam) and anti-Sca-1 (Abcam) antibodies were also used at 1:100 dilution. Filamentous actin was detected by using rhodamine phalloidin. Hoechst (10 μg/mL (Molecular Probes, Eugene, OR, USA) staining at 1:1000 dilution was used for the detection of cell nuclei. The cells were stained with antibodies at room temperature.

**Visualization.** A microscope “Axio Observer.Z1” (Carl Zeiss) with ×10, ×20 and ×40 (oil) lens was used to fluorescent microscopy. Confocal microscopy was performed by using the Leica TCS SP5 microscope with ×20 and ×40 (glycerol) magnification. Intensity of the corresponding fluorochrome served to establish a type of CSC, which generated a colony or CICS. In addition, it enabled one, for the first time, not only to reveal c-kit^+^- and Sca-1^+^- CSCs but also to identify an antigen phenotype of the host cell when CICSs are formed. Negative responses to Islet-1 antibodies indicate that CSCs of this type do not form CICSs in the myocardium of adult mammals. Furthermore, the lack of positive staining with antibodies to smooth muscle, endothelial cells, and fibroblasts demonstrated that intracellular development of c-kit^+^- and Sca-1^+^- CSCs occurs only in variously matured CMs.

**Time-lapse microscopy.** The images of the living cardiomyocytes were recorded at a magnification of ×40 (glycerol) for 7 days, at a rate of 1 frame per min (Zeiss Cell Observer SD, Carl Zeiss, Germany).

**Statistical analysis.** All of the data are expressed as the mean ± standard deviation. The statistical analyses were performed by using the SPSS 13.0 software package (SPSS Inc. Software, Chicago, IL, USA).

## 3. Results

**Figure 1:**
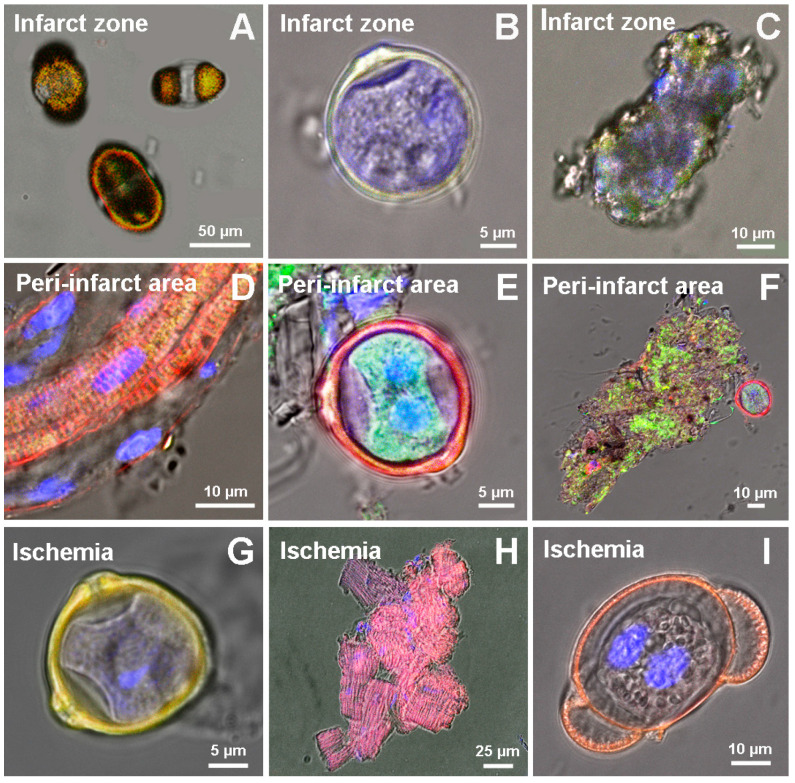
Cellular structures, identified in the suspension of the myocardial cells in adult rats two weeks after permanent coronary occlusion in the infarct zone (**A**–**C**), peri-infarct area (**D**–**F**), and after 40-min ischemia/reperfusion (**G**–**I**). Confocal and fluorescent microscopy: c-kit^+^-, Sca-1^+^-, and Islet-1^+^- CSCs (green); α-sarcomeric actinin (red); and nuclei (Hoechst 33342, blue).

Examination of the suspension of myocardial cells at two weeks both after permanent coronary occlusion and ischemia–reperfusion revealed an active involvement of CSCs in regenerative cardiomyogenesis. Along with colonies of fibroblasts and single uninjured CMs directly in the area of infarction (PCAO group), we observed remnants of ruptured encapsulated pre-existing CICSs (Figure 1A), newly formed immature encapsulated CICSs (Figure 1B), and small undifferentiated Sca-1^+^ CSC-derived colonies (Figure 1C). In the peri-infarct area, immature encapsulated CICSs (Figure 1E) and c-kit^+^ CSC-derived colonies (Figure 1F) were found, along with clusters of uninjured CMs (Figure 1D). In the MIRI group, not only uninjured CMs, but encapsulated CICSs of different maturity (Figure 1G), young small-sized newly formed CMs (Figure 1H), and ruptured capsules of formerly existing CICSs (Figure 1I) were identified.

**Figure 2:**
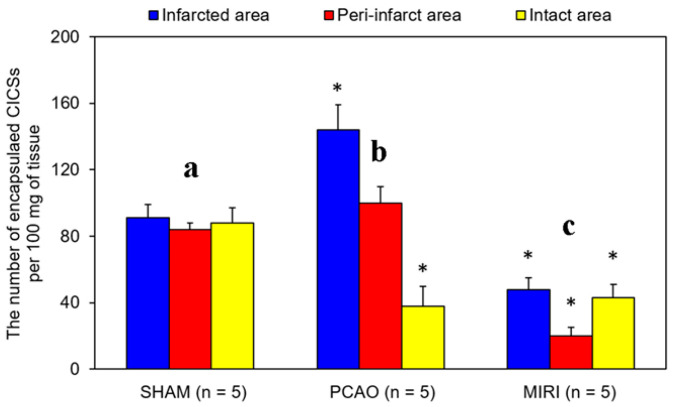
The average number of encapsulated CICSs in infarct, peri-infarct, and intact areas of the heart in experimental groups two weeks after surgery in the Control (**a**) 2 weeks after permanent myocardial infarction (**b**) and 40-min ischemia/reperfusion (**c**). The data are mean ± standard deviation. SHAM—sham-operated animals, PCAO—permanent coronary artery occlusion, MIRI—myocardial ischemia for 40 min with reperfusion; * *p* < 0.05 versus the respective value in SHAM group.

The data on the number of intact and ruptured encapsulated CICSs at various time points in all groups studied in three areas of the myocardium (infarction, peri-infarction, and remote to injury areas) are given in Figure 2. In the Controls (Figure 2a), the average numbers of encapsulated CICSs were not different in all three areas analyzed, amounting, on average, to 87.7 ± 3.5 encapsulated CICSs per 100 mg of myocardial tissue. Both infarction and ischemia–reperfusion significantly modulated this parameter. In the PCAO group (Figure 2b), the number of encapsulated CICSs exceeded that in the Controls by 1.5 times, reaching the value of 144.6 ± 15.3 per 100 mg of tissue in the infarct area. In peri-infarct area, the amount of encapsulated CICSs was comparable to that of the Controls (100.2 ± 10.5), but in the intact area it was significantly smaller than in the Controls (38.7 ± 12.5). In MIRI group, the number of encapsulated CICSs was significantly smaller in all areas analyzed (Figure 2c), averaging 48.4 ± 7.3, 20.3 ± 5.4, and 43.8 ± 8.6 in infarct, peri-infarct, and intact area, respectively.

**Figure 3:**
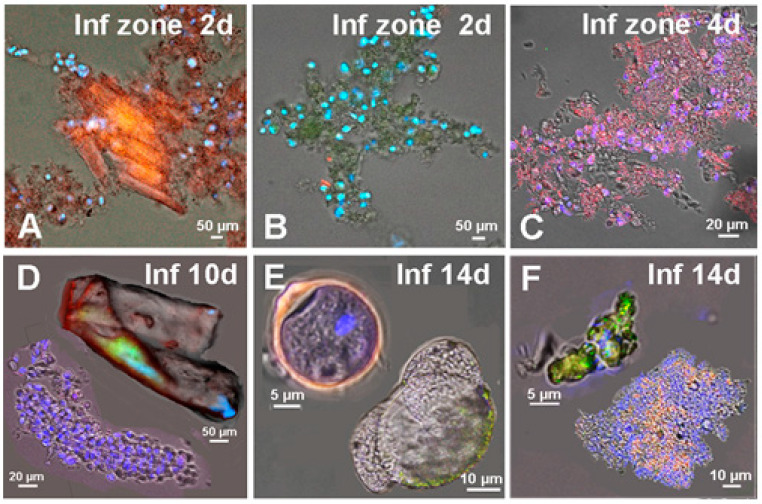
Variants of cellular structures revealed in the suspension of the myocardial cells in adult rats from the infarct area at different time points after permanent coronary occlusion. (**A**) Myocardial fragment with mature CMs and TACs; (**B**) accumulation of undifferentiated TACs, descendants of c-kit^+^- CSCs; (**C**) accumulation of cardio-positive TACs; (**D**) colony of fibroblasts (at the bottom) and dead CM (at the top); (**E**) immature c-kit^+^- CICS (at the top) and capsule of opened/ruptured CICS (at the bottom); (**F**) small colony of undifferentiated c-kit^+^- CSC (at the top) and large differentiated colony c-kit^+^- CSC (at the bottom). Confocal and fluorescent microscopy: c-kit^+^- CSCs (green), α-sarcomeric actinin (red), and nuclei (Hoechst 3342, blue).

Confocal and fluorescent microscopy of myocardial cell suspension in PCAO group revealed clusters of uninjured CMs surrounded by relatively large cardio-positive TACs (Figure 3A) and groups of undifferentiated TACs, formed by CSCs of c-kit^+^-type (Figure 3B) in infarct area at two days after PCAO. Four days after PCAO, fully mature TACs (Figure 3C), and 10 days after PCAO, necrotic CMs, and colonies of fibroblasts (Figure 3D) were found. At two weeks after PCAO, we observed immature encapsulated CICSs and empty shells of ruptured CICSs (Figure 3E) in the infarct area, as well as small undifferentiated and larger, more differentiated colonies formed by CSCs of c-kit^+^-type (Figure 3F), suggesting re-initiation of cardiomyogenesis in the infarct area, and thus confirming the involvement of c-kit^+^ CSCs in myocardial regeneration.

**Figure 4:**
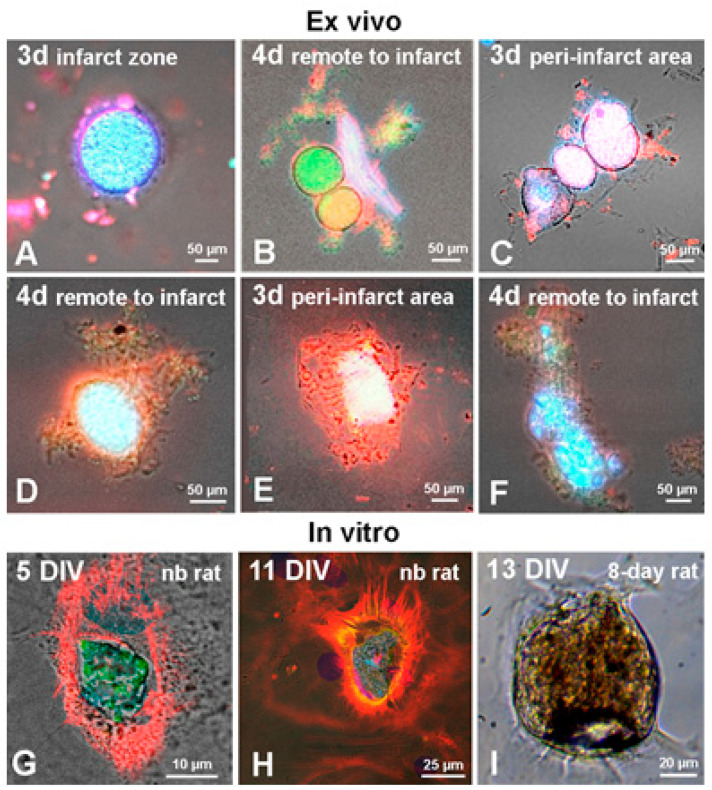
Variants of non-encapsulated CICSs in the suspension of the myocardial cells in adult rats after permanent coronary occlusion (ex vivo) and in the culture of the myocardial cells in rats (in vitro). Ex vivo: Cellular structures from the infarct zone (**A**), peri-infarct area (**C**,**E**) and the area remote from the infarct zone (**B**,**D**,**F**), demonstrating intracellular development of CSCs with formation of non-encapsulated CICSs. A—free vacuole with TACs of c-kit^+^- type; B—disintegrating CM, releasing 2 vacuoles with c-kit^+^- TACs of various inner maturity; C—3 vacuoles with c-kit^+^- TACs inside, located in the cytoplasm of one CM; D, E—vacuoles with c-kit^+^- TACs inside mature CMs; F—mature CM with abundant c-kit^+^- TACs inside. In vitro: non-encapsulated CICSs in newborn rats on day 5 in vitro (DIV) –with c-kit^+^ CSCs and rhodamine phalloidin (**G**) and on DIV 11 with Islet-1^+^ CSCs and cardiac myosin (**H**). Non-encapsulated CICSs in 8-day old rat on DIV 13 (light microscopy—(**I**)). Confocal and fluorescent microscopy: c-kit^+^- and Islet-1^+^- CSCs (green), α-sarcomeric actinin, rhodamine phalloidin and cardiac myosin (red), nuclei (Hoechst 33342, blue).

**Figure 5:**
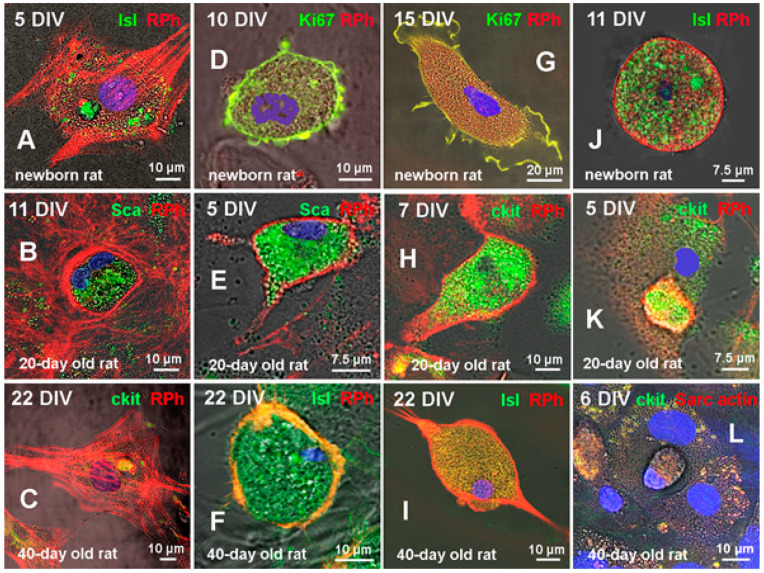
Confocal microscopy of cellular structures revealed when culturing the myocardial cells of variously aged rats. Newborn rats: on day 5 in vitro (DIV)—(**A**); on DIV 10—(**D**); on DIV 15—(**G**) and on DIV 11—(**J**). Twenty-day-old rats on DIV 11—(**B**); on DIV 5 (**E**,**K**); and on DIV 7—(**H**). Forty-day-old rats on DIV 22—(**C**,**F**,**I**) and on DIV 6 (**L**). Confocal microscopy using proliferation marker Ki67 (green); rhodamine phalloidin (red); of antibodies to Islet-1, c-kit, and Sca-1 CSCs (green); sarcomeric actin (red), and nuclei (Hoechst 33342, blue).

In addition, in the early post-infarction period we identified another type of CICSs, which were sphere-shaped, averaged 200 µm in diameter (Figure 4A), and contained multiple small cells bearing c-kit and stained with α-sarcomeric actinin. Additional qualitative characterization of these structures is provided in Figure 4B–F (Supplementary Materials Video S1). It is apparent that these structures are localized inside mature CMs, representing distinct type of CICSs, which are morphologically different from previously described encapsulated CICSs [15,16]. Taking into consideration the difference of these newly identified structures from earlier described CICSs, we suggest the term “non-encapsulated CICSs” in order to designate them. It was found that one large CM can contain several such non-encapsulated CICSs. This suggests that a single host CM can be penetrated by several stem cells, each of which will multiply inside a separate vacuole (Figure 4B,C). Upon disintegration of the host CM, these intracellular structures are distributed within the myocardium, and, in our model, they appeared in the medium as membrane-bound spheres containing many TACs inside (Figure 4A). However, when only a single CSC enters the mature CM and starts to proliferate inside, the different scenario may take place. The photos presented in Figure 4D–F demonstrate that the size of intracellular vacuole can progressively increase, and, in some cases, it can almost entirely occupy the internal space of large CM (Figure 4F). Identical structures involving Islet-1^+^ and c-kit^+^ CSCs were also detected during in vitro experiments (Figure 4G,H). A photo of the sphere-shaped structure that was formed over 13 days of culturing of 8-day-old rat myocardial cells shows that there are multiple small cells inside the structure with D = 80 µm (Figure 4I). Our comparison of the morphology of intracellular structures revealed ex vivo (Figure 4) and in vitro (Figure 5) enables not only a description of CICSs at different stages, but also makes it possible to discuss the potential conditions contributing to the formation of particular types of CICS.

For instance, microphotographs in the left vertical row in Figure 5A–C demonstrate the presence of three types (Islet-1^+^, Sca-1^+^, and c-kit^+^) of CSCs inside CMs of newborn (A), 20-day-old (B), and 40-day-old (C) rats. Here, it should be noted that CSCs can either be positioned close to the nucleus (or between nuclei) of the host cell, being in this case inside an individual vacuole (Figure 5A,C), or co-localize with the nucleus of the host cell being separated from the cytoplasm by the sarcolemmal membrane (Figure 5B). The first variant of CICS formation was previously described by us in the culture of neonatal myocardial cells [15]. It was shown that CSC penetrates CM through invagination of the sarcolemma, being localized close to the host-cell nucleus, but inside the separate vacuole bound by a fragment of inverted sarcolemma. Dynamic observation of this CICS type in vitro has shown that CSCs are proliferating inside the vacuole, producing a population of TACs. More advanced stages of CICS development are characterized by the growth of vacuole in diameter, which is accompanied by the formation of dense actin-positive capsule around it inside the host cell. We supposed that the capsule is a result of compactization of intracellular actin. This idea is supported by the notion that actin filaments make up the cytoskeleton of any immature cell, neonatal cardiomyocyte including [15]. Hence, we assumed that formation of encapsulated CICSs takes place when CSCs develop inside immature CM or TAC, which, according to our data, are formed during myocardial self-renewal throughout the entire lifespan. Similar localization of CSC-filled vacuole was also observed in the culture of neonatal myocardial cells (Figure 5A), and in the culture of cardiac cells of 40-day-old rats (Figure 5C). It is worth noting at this point that all CMs presented in these figures demonstrate a clearly defined actin cytoskeleton.

The non-encapsulated CICS shown in Figure 5B exhibits a co-localization of Sca-1^+^ CSC with two nuclei of the host cell inside the area limited by the actin-positive membrane, which may evidence for intermediate maturity of this CM. The structures of this type are also presented in Figure 5D–I. Non-encapsulated CICSs in Figure 5D–F are likely corresponding to a somewhat earlier stages of their development, while Figure 5G–I shows more advanced stages of non-encapsulated CICS development. In turn, Figure 5J demonstrates large non-encapsulated CICS formed in the culture of cardiac cells of newborn rats. In this case, the lack of capsule may indicate that Islet-1^+^ CSC developed not inside the TAC, but inside a more mature neonatal CM. Besides, CM of 20-day-old rats in Figure 5K is not enriched in actin myofilaments but contains non-encapsulated CICS with small vacuole filled with descendants of c-kit^+^ CSC. In turn, Figure 5L presents an early stage of non-encapsulated CICS formation in the culture of 40-day-old rat myocardial cells. It is seen that the vacuole located close to the host cell nucleus incorporates only single CSC of c-kit^+^ type.

Additional staining for rhodamine phalloidin confirmed the lack of actin cytoskeleton in CMs bearing various stages of non-encapsulated CICSs in newborn, 20-day-old and 40-day-old rats (Figure 5D–I). Morphological characteristics of more mature CMs without actin cytoskeleton (Figure 5G–I) allow us to suggest that CSCs in differentiated CMs develop inside the vacuole, located in the host-cell sarcolemma between actomyosin complex layers, which is analogous to variants D–F in Figure 4.

**Figure 6:**
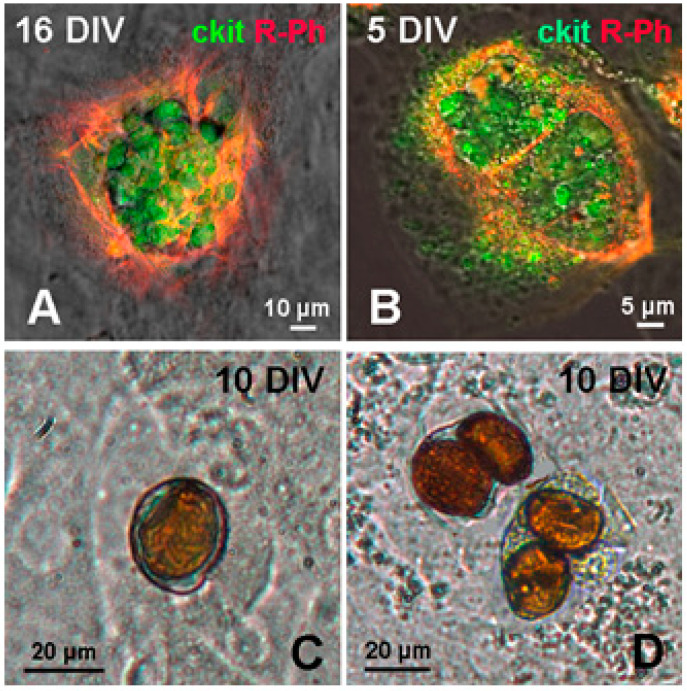
Variants of CICSs in the culture of the neonatal myocardium cells of rats (in vitro). (**A**,**B**) confocal microscopy; (**C**,**D**) light microscopy (x40).Upper row: (**A**) non-encapsulated CICS on day 16 in vitro (DIV), formed with CSC of c-kit^+^ type; (**B**) non-encapsulated CICS on DIV 5 with two c-kit^+^- vacuoles inside. Bottom row: (**C**) encapsulated CICS; (**D**) 2 encapsulated CICSs with two c-kit^+^- capsules inside each. Confocal microscopy using antibodies to c-kit CSCs (green) and rhodamine phalloidin (red).

The data presented in Figure 6 illustrate the morphology of two variants of CICSs observed after penetration of mature CM with either one (Figure 6A,C) or two CSCs (Figure 6B,D). According to our hypothesis, when CSC enters immature CM, it starts to divide inside a single vacuole, thereby increasing in volume and causing compression of actin cytoskeleton around, eventually forming an individual dense capsule with openings. When just one CSC enters CM, one capsule is formed (Figure 6C), whereas, after the penetration of two CSCs, each of them produces an individual capsule (Figure 6D). In the case of non-encapsulated CICS formation due to the entry of one, two or more CSCs, each of them will proliferate inside an individual vacuole, surrounded by actin-positive membrane (Figure 6A,B). However, because of the depolymerized state of the actin in differentiated CM, no capsule is formed.

According to our in vitro data, the proportion of CICSs in the myocardium is about 0.001–0.002%. Despite this negligible amount, a question arises as to how these structures affect myocardial function and whether they impede cardiac muscle contraction. Our analysis of the formation and development of encapsulated CICSs in the primary culture has enabled us to establish that immature CMs, which are the host cells for CSCs, maintain viability over the entire course of intracellular CSC development, dying only during CICS rupture, accompanied by the release of ~200 TACs [15]. Moreover, at least in the culture of the neonatal myocardial cells the presence of CSCs inside the CM does not interfere with the process of contraction of the latter (Supplementary Materials Video S2). In turn, pre-existing CICSs formed in the in vivo settings, upon plating in the culture, are becoming attached to the substrate and display viability identical to that of the other cells. While lacking their own contractile activity, they do not impede contractions of the neighboring CMs (Supplementary Materials Videos S3 and S4).

**Figure 7:**
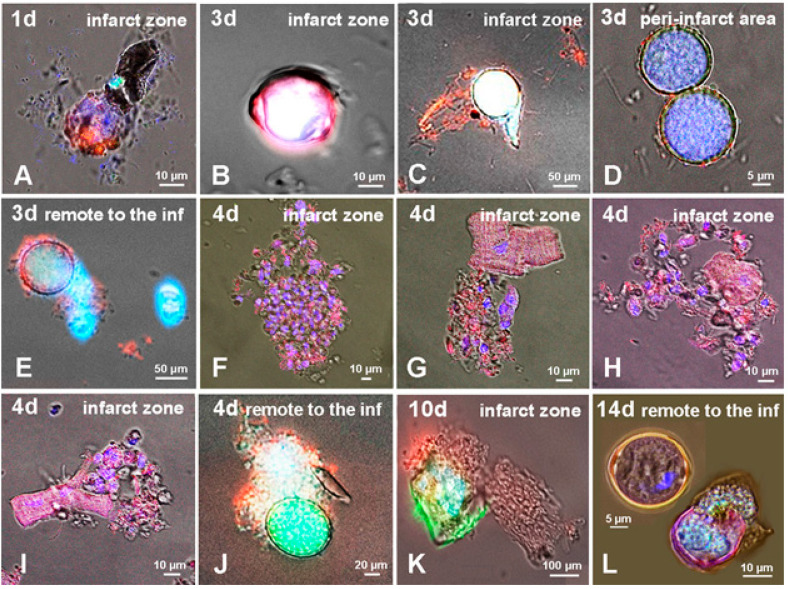
Cellular structures in the infarct area (**A**–**C**,**F**–**I**,**K**), peri-infarct area (**D**), and the area remote from the infarct (**E**,**J**,**L**). (**A**,**B**,**L**) Encapsulated CICSs. (**C**–**E**,**J**,**K**) Non-encapsulated CICSs. (**A**,**L**) (bottom) Release of TACs from encapsulated CICSs. (**G**–**I**) Mass egress of TACs from mature CMs. (**F**) Population of TACs inside the mucin mass after exiting CICS. Confocal and fluorescent microscopy with staining: c-kit^+^- CSCs (green), α-sarcomeric actinin (red), and nuclei (Hoechst 33342, blue).

When analyzing the suspension of myocardial cells from the infarct area in the first days after PCAO, it was found that some pre-existing encapsulated CICSs ruptured on the day 1 after coronary occlusion (Figure 7A). Three days after PCAO, free spherical structures with a large number of TACs inside were found in the suspension (Figure 7D), as well as ruptured intracellular vacuoles localized in the sarcoplasm of mature CMs (Figure 7C) and free spherical vacuoles (Figure 7E). On the fourth day after PCAO, disintegration of mature CMs releasing numerous well-differentiated TACs (Figure 7G–I) was recorded in the infarct area, thus completing a scenario of intracellular CSC development presented in Figure 4D–F. In turn, the release of non-encapsulated CICSs with immature TACs inside (Figure 7J) was observed predominantly in the intact area remote from the infarct. The release of c-kit^+^ TACs from non-encapsulated CICSs present inside mature CMs was evident even at 10 days after PCAO (Figure 7K).

These data, along with detection of encapsulated CICSs and ruptured CICSs two weeks after PCAO (Figure 7L), provide the basis for the suggestion that CSC-mediated regenerative cardiomyogenesis is initiated in the myocardium immediately after the onset of myocardial ischemia and continues throughout the entire period of our observations, that is, at least for 14 days post-MI. At present, we have insufficient data to explain the phenomenon of dominating release of mostly undifferentiated TACs from non-encapsulated CICSs in the first 3 days after PCAO (Figure 7C–E). Similarly, it is not clear why this is changed into the release of more differentiated TACs into the myocardium (Figure 7F–I) at a later phase (≥4 days) after the infarction. We can only assume that both encapsulated and non-encapsulated CICSs with poorly differentiated TACs inside are undergoing early rupture due to greater sensitivity to hypoxia and pH reduction at the acute stage of ischemia. If this is true, the key proximal question is the estimation of the survival rate of TACs released from encapsulated and non-encapsulated CICSs in the damaged area. In the case of their massive death, the myocardium is deprived of the significant number of TACs, which are in principle able to replace lost mature CMs. However, the release of highly differentiated TACs from mature CMs (Figure 7G–I) directly into the infarct area inspires some optimism, since the integration of these cells into the myocardium may be considered as a viable target in therapeutic cardiomyogenesis.

## 4. Discussion

One of the main findings of the present study is that the number of encapsulated TAC-containing CICSs in the myocardium is changed after myocardial ischemia. In particular, permanent coronary ligation has resulted in a significant increase in the amount of encapsulated CICSs in infarct area, while it has not been different from controls in peri-infarct area and has become smaller in intact myocardium compared to control animals. In contrast, transient ischemia followed by reperfusion was associated with decreased amount of CICSs in all myocardial areas studied. According to our data obtained in the primary culture of neonatal myocardial cells, the complete lifecycle of encapsulated CICS from the moment of CSC penetration into CM to CICS rupture and TAC release takes about 20–25 days [15]. When analyzing the occurrence of encapsulated CICSs in the hearts of animals 2 weeks after PCAO or MIRI, we observed the net result of two different processes: (1) the development and rupture of pre-existing encapsulated CICSs of various maturity, which were formed prior to surgery; and (2) the evolution of encapsulated CICSs, which were formed after surgical intervention. Based on this, we can explain a 1.5-fold increase in the number of encapsulated CICSs in the infarct area by the fact that ischemia accompanied by hypoxia, inflammation, and acidification of the tissue adversely affects the function of CSCs and immature TACs, inhibiting the formation of colonies, but, at the same time, stimulates intracellular development of CSCs in CMs of different levels of maturity. This is in good agreement with the results of our in vitro experiments that show a 5–10-fold increase in the number of encapsulated CICSs after induction of hypoxia and acidification of the culture medium [16]. The number of encapsulated CICSs in peri-infarct area after permanent coronary ligation was not different from control, demonstrating the existence of certain equilibrium between the rate of rupture of pre-existing CICSs and the rate of formation of new encapsulated CICSs, as well as formation of CSC-derived colonies. The decreased number of encapsulated CICSs in intact heart area may be accounted for decreased formation of new encapsulated CICSs in the area distant from the infarction, since the cardiomyogenesis in this normal tissue mainly occurs by the proliferation and differentiation of CSCs within the colonies, but not through the intracellular development of CSCs. It is likely that the formation of colonies in the area of intact myocardium may even be more feasible than the formation of CICSs, since the differentiation of TACs inside colonies occurs faster, allowing them to establish contractile behavior after only 10–15 days [14]. Of note, the number of encapsulated CICSs in infarct and intact areas in animals subjected to transient ischemia was not different from that in intact area in PCAO group, which lends support to the hypothesis that the predominant pathway of cardiomyogenesis is determined by the local environment. Given that formation of colonies and encapsulated CICSs provide equal contribution to cardiomyogenesis in healthy myocardium [15]**,** one can suppose that ischemia–reperfusion can shift the equilibrium towards more intense formation of new CMs within the colonies, which would reduce the number of encapsulated CICSs in the settings of constant pool of resident CSCs.

In this study, a new type of CSC-containing CICSs was identified and described for the first time in heart samples obtained in the early post-infarct period. Based on the characteristics of the envelope of these structures, we have termed them non-encapsulated CICSs. According to our data [15], encapsulated CICSs could be formed only upon penetration of CSC into an immature CM with an actin cytoskeleton. In this case, multiple rounds of division of the original CSC cause a progressive increase in the size of the vacuole, thereby displacing the actin filaments to the periphery of the cell. Compression of actin promotes its polymerization, resulting in the formation of a capsule located between the membranes of the vacuole and the host cell. It is known that actin is able to polymerize, ensuring the process of cell motility [18,19,20]. However, when CSC enters the mature CM, no actin capsule is formed, because actin is a part of the actomyosin complex providing building blocks for sarcomeres and CM contraction.

In our opinion, the abovementioned characteristics of two types of CICSs, that is, encapsulated vs. non-encapsulated CICSs, are responsible for the differences in maturity of TACs which develop inside them. Of note, the greater extent of TAC maturity was observed in non-encapsulated CICSs, which might have been accounted for by the higher level of differentiation of host cell and easier availability of humoral factors diffusing to the membrane-enveloped CSCs. In contrast, less differentiated TACs were released from encapsulated CICSs, which is paralleled by lower differentiation status of the host cell and poor diffusion of paracrine factors stimulating differentiation through the thick capsule. Indeed, according to our confocal microscopy data, at least 200 poorly differentiated TACs with L = 10–12 µm are formed inside the capsule during the proliferation of CSC, which, upon destruction of CICSs, are able to divide and differentiate, thereby forming new CMs [15]. Non-encapsulated CICSs release larger TACs with L ˃ 12–18 µm, characterized by lower proliferative potential and greater extent of differentiation than TACs from encapsulated CICS. Therefore, the latter subtype of TACs might replenish the population of precardiomyocytes, which, in parallel with differentiation, can grow, reaching L = 30–60 µm. The compact structure, size range, and weak expression of stemness markers are all criteria for allocating these cells to the population of young CMs formed inside non-encapsulated CICSs. Moreover, we think that these data contradict those of Senyo et al. [10,11], who explain the appearance of small cardiac cells in post-infarction myocardium by the division of mature CMs. Our data show that both types of CICSs could have underwent rupture in the infarct area in the first days after permanent coronary ligation. Indirect evidence indicates that the massive rupture of encapsulated CICSs takes place on the next day after PCAO, while non-encapsulated CICSs are massively destroyed with the release of numerous differentiated TACs four days after infarction. The main unanswered question now is the origin of stimulus causing CICS rupture after PCAO. We presume that this event could be linked to physicochemical changes typical of acute ischemia, including hypoxia and acidosis, both of which could lead to destabilization of the membrane surrounding CICSs. However, the fact that differentiated TACs exit CMs directly into the infarct area, while vacuoles with immature TACs are released from non-encapsulated CICSs in peri-infarct and intact area, suggests that these variants reflect potential mechanisms of regeneration process. It can be supposed that myocardial regeneration in the infarct area requires the cells, which are able to quickly replenish lost CMs, namely precardiomyocytes (or differentiated TACs), while immature TACs can be employed in less damaged myocardial segments. Although many important questions lie ahead, we could conclude that adult mammalian myocardium is capable of inducing self-regeneration process after permanent coronary occlusion and ischemia–reperfusion, which involves TACs—of c-kit^+^- and Sca-1^+^ CSCs. Formation of new CMs through proliferation and differentiation of CSCs within colonies is blocked after infarction and restored only 10–14 days after, the cardiomyogenesis in the early post-infarction period occurs by using population of TACs, which develop inside cardiac cells of different levels of maturity with formation of encapsulated and non-encapsulated CICSs.

Moreover, our previous study demonstrated that increased CO_2_ percentage (10–13%) stimulated the intracellular development of CSCs, enhancing the numbers of CICSs by 5–10 times in cell culture compared to the control [16]. These data suggest that hypoxia may represents one of the factors regulating the balance between formation of colonies and CICSs. Hypothetically, the effects of hypoxia on CSCs could be mediated by the activation of hypoxia-inducible factor-1 (HIF-1), which controls the number of genes involved in adaptation to hypoxia [21]. It is also known that hypoxia can stimulate proliferation and differentiation of embryonic stem cells (ESCs) [22]. Hypoxic preconditioning of both ESCs and induced pluripotent stem cells (iPSCs) prior to their transplantation into ischemic myocardium enhanced the therapeutic potential of the procedure [23]. Molecular mechanism(s) of prosurvival effect of 6-h hypoxic preconditioning on c-kit^+^ CSCs is mediated by increased expression of Pim-1 kinase and concomitant reduction in pro-apoptotic factors [24]. Therefore, improved understanding of molecular mechanisms underlying hypoxia-mediated signaling in CSCs, both giving rise to colonies and CICSs, may contribute to development of new pharmacological therapies targeting the process of cardiomyogenesis.

We suppose that the data presented are essential for developing new strategies for myocardial regeneration after irreversible injury. Although it is not easy to envisage the potential clinical applications of the phenomena described, we did our best to provide certain relevant speculations. According to our findings, it is felt that TACs might become the crucial cellular target for cardioregenerative therapy in the future. There are several potential ways to harness regenerative potential of TACs. First, one cannot exclude the possibility that TACs will be directly transplanted in the damaged heart by using 3D NOGA endocardial mapping system and electromagnetically guided percutaneous intramyocardial therapy. It is also conceivable that TACs will be subjected to genetic modification aimed at boosting of their cardiomyogenic potency and/or enhancing their survival in the harsh ischemic environment prior to intramyocardial administration. Second, the endogenous pool of TACs could be activated by means of their pharmacological stimulation with growth factors, microRNAs, and low-molecular-weight drugs. These stimulating factors could be packed into membrane vesicles or bound with other transporting vehicles, which might increase their targeted delivery into injured myocardium after systemic administration. Third, there might be an opportunity to “switch” from one pathway of cardiomyogenesis to another, thereby providing optimal contribution of colony-derived cardiomyocytes and those produced within encapsulated and non-encapsulated cell-in-cell-structures. For instance, within the first days after infarction, the main cellular target for myocardial regeneration would be the population of immature TACs released from encapsulated CICSs. The approaches aimed at enhanced survival of these cells, as well as at their augmented proliferation and differentiation, could potentially contribute to formation of new functionally competent CMs. During the more protracted stage of infarction, namely starting from the 4th day, the most promising endogenous cell population for heart regeneration would be TACs developing inside mature CMs, that is, in non-encapsulated CICSs. Future studies, no doubt, will shed light on the molecular mechanisms of CSC-mediated myocardial self-renewal and regeneration.

## Data Availability

Not applicable.

## Supplementary Materials

The following are available online at https://www.mdpi.com/article/10.3390/life11080723/s1. Video S1: The presence of a CSC-vacuole inside the neonatal rat cardiomyocyte. Video S2: The presence of CSC-capsule inside the cardiomyocyte of 6-day-old rat. Video S3: Confluent monolayer of contracting cardiomyocytes (28 bpm). Video S4: Vacuole with a dense membrane inside the mature cardiomyocyte of an adult rat (c-kit, green; α-actinin, red; and nuclei, blue).

## Abbreviations

CSCs	cardiac stem cells
TACs	transitory amplifying sells
CICSs	cell-in-cell structures
CM	cardiomyocyte
SHAM	sham-operated animals
PCAO	permanent coronary artery occlusion
MIRI	myocardial ischemia for 40 min with reperfusion
